# Association between disorganization of the retinal inner layers and capillary nonperfusion area in patients with retinal vein occlusion

**DOI:** 10.5935/0004-2749.20200093

**Published:** 2024-02-11

**Authors:** Yasin Sakir Goker, Cemile Ucgul Atılgan, Kemal Tekin, Hasan Kızıltoprak, Pinar Kosekahya, Gokhan Demir, Deniz Kılıc

**Affiliations:** 1 Ankara Ulucanlar Eye Training and Research Hospital, Ankara, Turkey; 2 Van Ercis State Hospital, Van, Turkey; 3 Ophthalmology Department, Bingol Woman’s Health and Children’s Hospital, Bingol, Turkey; 4 Beyoglu Eye Training and Research Hospital, Istanbul, Turkey; 5 Kayseri City Hospital, Kayseri, Turkey

**Keywords:** Retinal vein occlusion/diagnosis, Macular edema/ physiopathology, Retina/pathology, Capillaries/pathology, Fovea centralis, Retinal vessels/pathology, Fluorescein angiography, Tomography, optical coherence, Oclusão da veia retiniana/diagnóstico, Edema ma cu lar/fisiopatologia, Retina/patologia, Capilares/patologia, Fó, vea central, Vasos retinianos/patologia, Angiofluoresceinograf ia, To mografia de coerência óptica

## Abstract

**Purpose:**

To determine the correlation between the extent of disorganization of the retinal inner layers (a parameter of spectral domain optical coherence tomography) and optical coherence tomography angiography parameters in eyes with center-involved macular edema associated with retinal vein occlusion.

**Methods:**

This retrospective observational study included 34 eyes of 34 patients with newly diagnosed macular edema associated with retinal vein occlusion and evidence of center-involved macular edema. Optical coherence tomography angiography and spectral domain optical coherence tomography were evaluated after resolution of the macular edema. Disorganization of the retinal inner layers was determined via spectral domain optical coherence tomography and optical coherence tomography angiography parameters, including foveal avascular zone area in the superficial capillary plexus and capillary nonperfusion areas, foveal avascular zone area in full retinal vasculature, foveal avascular zone perimeter, acircularity index of the foveal avascular zone, and foveal density.

**Results:**

The mean disorganization of the retinal inner layers extent was 512.72 ± 238.47 microns, and the mean capillary nonperfusion area was 4.98 ± 2.85 mm^2^. There was a positive correlation between the extent of disorganization of the retinal inner layers and capillary nonperfusion area (p<0.001, r=0.901). Greater extent of disorganization of the retinal inner layers and the capillary nonperfusion area was correlated with wider foveal avascular zone area (p=0.014 and p=0.036, respectively) in the superficial capillary plexus and decreased foveal density (vessel density in 300 microns around the foveal avascular zone) (p=0.031 and p=0.022, respectively). These parameters were also correlated with decreased vessel density in both the superficial capillary plexus and deep capillary plexus in the parafoveal and perifoveal regions (p<0.05 for all).

**Conclusions:**

Disorganization of the retinal inner layers appears to be a correlated biomarker of capillary ischemia in retinal vein occlusion. The extent of disorganization of the retinal inner layers was strongly correlated with the capillary nonperfusion area. This may support the notion that the extent of disorganization of the retinal inner layers can be used as an easily obtainable and crucial surrogate marker of capillary ischemia.

## Abstract (pt)

**Objetivo:**

Determinar a correlação entre a extensão da desorganização das camadas internas da retina, que constitui um parâmetro da tomografia de coerência óptica de domínio espectral, e os parâmetros da angiografia por tomografia de coerência óptica em olhos com edema macular com envolvimento central associado à oclusão da veia retiniana.

**Métodos:**

Este estudo retrospectivo observacional incluiu 34 olhos de 34 pacientes com edema macular recém-diag nosticado associado à oclusão da veia retiniana e com evidência de edema macular com envolvimento central. Após a resolução do edema macular, foram avaliadas a tomografia de coerência óptica de domínio espectral e a angiografia por tomografia de coerência óptica. A desorganização das camadas internas da retina foi determinada através de parâmetros da tomografia de coerência óptica de domínio espectral e da angiografia por tomografia de coerência óptica, incluindo a área da zona avascular foveal no plexo capilar superficial e nas regiões sem perfusão capilar, a área da zona avascular foveal na vascularização total da retina, o perímetro da zona avascular foveal, o índice de não circularidade da zona avascular foveal e a densidade foveal.

**Resultados:**

A extensão média da desorganização das camadas internas da retina foi de 512,72 ± 238,47 µm e a área média da região sem perfusão capilar foi de 4,98 ± 2,85 mm^2^. Houve uma correlação positiva entre a extensão da desorganização das camadas internas da retina e a área da região sem perfusão capilar (p<0,001, r=0,901). Maior extensão da desorganização das camadas internas da retina e da região sem perfusão capilar correlacionaram-se a uma área maior da zona avascular foveal (respectivamente, p=0,014 e p=0,036) no plexo capilar superficial e a uma menor densidade foveal (a densidade vascular nos 300 µm à volta da zona avascular foveal; respectivamente, p=0,031 e p=0,022), e também se correlacionaram a uma menor densidade vascular tanto no plexo capilar superficial como no profundo, nas regiões parafoveal e perifoveal (p<0,05 em todas as correlações).

**Conclusão:**

A desorganização das camadas internas da retina parece ser um biomarcador correlacionado com a isquemia capilar na oclusão da veia retiniana. O fato de que a extensão dessa desorganização se correlacionou fortemente com a área sem perfusão capilar sugere o uso da extensão da desorganização das camadas internas da retina como um marcador substituto de isquemia capilar, sendo este um marcador importante e facilmente obtido.

## INTRODUCTION

Retinal vein occlusion (RVO) is the second most common retinal vascular disease causing macular edema (ME) and an important cause of visual deterioration^(1^,^2)^. Blocked venous drainage may induce an increase in the permeability of vessels and initiate a breakdown of the blood-retinal barrier. Eventually, cystoid ME, capillary nonperfusion (CNP), and ischemia occur. Ischemia is a crucial parameter in the prognosis of the disease. Traditionally, fundus fluorescein angiography (FFA) has been used to assess ischemia in these patients. Optical coherence tomography angiography (OCTA) is a noninvasive imaging technique that uses motion contrast imaging to obtain high-resolution volumetric blood flow information and generate angiographic images^(3)^. It also allows us to quantitatively measure the CNP area and the foveal avascular zone (FAZ).

Spectral domain optical coherence tomography (SD-OCT) is a reliable, high-resolution imaging technique that allows us to evaluate the retinal anatomy and quantify different prognostic parameters in patients with RVO, diabetic retinopathy, and uveitic cystoid ME^(4^-^6)^. Central macular thickness and the disorganization of the retinal inner layers (DRIL) are examples of these prognostic parameters^(4^-^6)^.

The aim of the study was to determine the correlation between the extent of SD-OCT parameter DRIL and OCTA parameters, including the FAZ area, CNP area (non-flow), and vessel density, in both the superficial capillary plexus (SCP) and deep capillary plexus (DCP) in eyes with center-involved ME associated with RVO (RVO-ME).

## METHODS

### Study population and design

This retrospective observational study was performed at the Ulucanlar Eye Research and Training Hospital (Ankara, Turkey). The study was performed according to the tenets of the Declaration of Helsinki and approved by the local ethics committee.

Consecutive cases of branch RVO (BRVO), examined between September 2017 and February 2018 by two retina specialists (Y.S.G. and C.U.A.) at the retina department of a tertiary referral center, were enrolled in this study. BRVO was determined by the presence of retinal vein dilatation, retinal edema, or superficial or deep hemorrhages confined to a focal region in the retina corresponding to a specific arteriovenous crossing. Inclusion criteria comprised newly diagnosed RVO-ME patients with evidence of center-involved ME. Exclusion criteria included substantial media opacity, axial length >24 and <22 mm, and intraocular operation within 12 months prior to study enrollment or during the follow-up period. Eyes with ocular comorbidities (e.g., retinal artery occlusion, uveitis, ME from any other cause, or vitreous traction) were excluded from the study. Patients initially underwent a visual acuity examination with a complete preliminary ocular examination, FFA, and SD-OCT scan (Spectralis; Heidelberg Engineering Inc., Heidelberg, Germany). Subsequently, they received monthly intravitreal injections of ranibizumab for 3 months as a loading dose, followed by an additional visual acuity examination, ocular examination, and SD-OCT and OCTA scans. Retreatment with ranibizumab was performed for relapses on a *pro re nata* basis. OCTA and SD-OCT analyses were performed after resolution of ME at the same visit.

### Data collection

The medical files of all eligible patients were reviewed, and the following data were collected: age, sex, involved eye, preliminary best-corrected visual acuity (BCVA), and BCVA following injections. BCVA was recorded in Snellen units and converted to a logarithm of the minimum angle of resolution (logMAR) for statistical analysis.

### SD-OCT measurements

**Figure 1:**
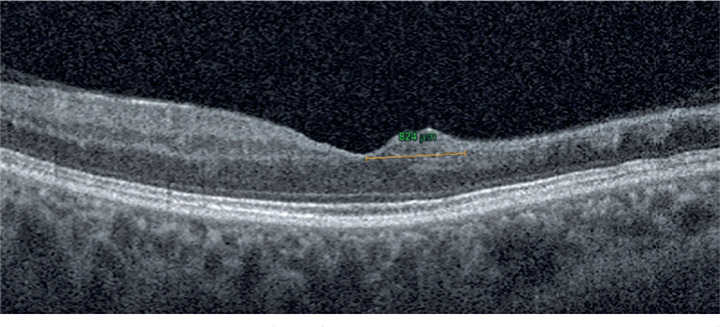
Disorganization of the retinal inner layers (DRIL) in the fovea was defined as the horizontal extent in microns for which any boundaries between the ganglion cell-inner plexiform layer complex, inner nuclear layer, and outer plexiform layer were indistinguishable. The DRIL extent of this patient was calculated as 824 microns.

Seven horizontal scans through the fovea and a 120-µm B-scan spacing were performed on the high-resolution mode of SD-OCT (Spectralis; Heidelberg Engineering) by a technician. DRIL was defined as the horizontal extent in microns for which any boundaries between the ganglion cell-inner plexiform layer complex, inner nuclear layer, and outer plexiform layer were indistinguishable^(7)^. Measurement of the DRIL extent through the fovea was retrospectively performed by two masked observers (Figure 1). The average of the measurements was used to derive a global DRIL measurement for the eye.

### OCTA measurements

All OCTA scans were performed by a single retina specialist (Y.S.G.) using the AngioVue software of the RTVue XR Avanti (Optovue Inc., Fremont, CA, USA). This system uses a split-spectrum amplitude-decorrelation angiography algorithm and operates at 70,000 A-scans per second to acquire OCTA volumes consisting of 400 × 400 A-scans. All scans of each eye measured 6 mm × 6 mm.

**Figure 2:**
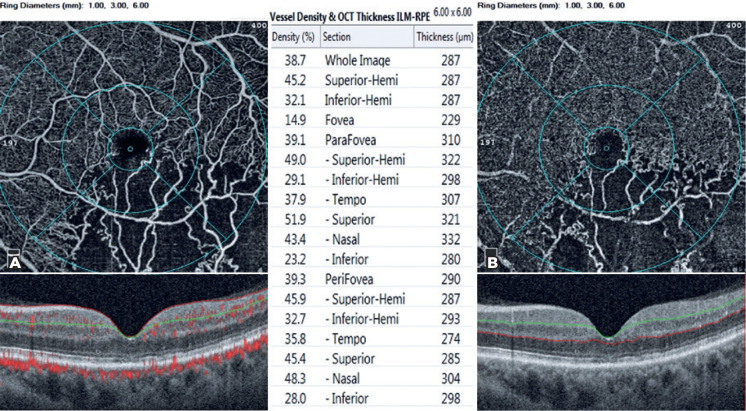
Density assessment tool of optical coherence tomography angiography (OCTA) (above) and B-scan of the fovea (below). A) Segmentation was between the internal limiting membrane and the inner plexiform layer in the superficial capillary plexus. B) Segmentation was between the inner nuclear layer and the outer plexiform layer in the deep capillary plexus.

The software (Version 2017.1.0.151) automatically inserted three fovea-centered circles on the macula. The density of the foveal zone vessel was defined as the area of the small circle, with a diameter of 1 mm. The density of the parafoveal zone vessel was defined as the area of the middle circle, with a diameter of 3 mm. The density of the perifoveal zone vessel was defined as the area of the outer circle, with a diameter of 6 mm. In addition, the parafoveal zone was automatically divided into four equal quadrants (i.e., temporal, nasal, inferior, and superior) and two equal hemispheres (i.e., superior and inferior) (Figure 2).

**Figure 3:**
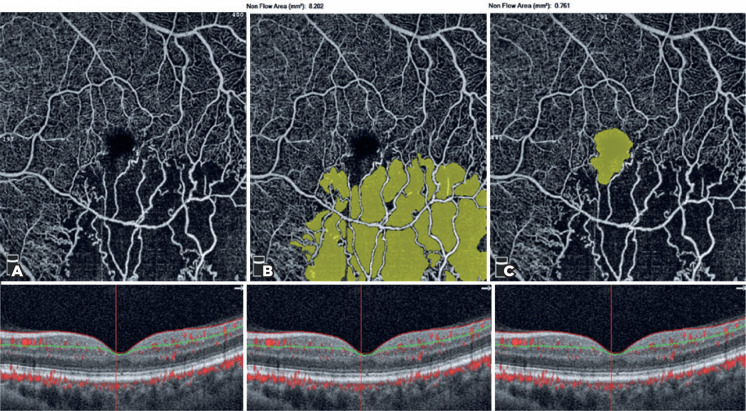
Non-flow assessment tool of optical coherence tomography angiography (OCTA) (above) and B-scan of the fovea (below). Segmentation was between the internal limiting membrane and the inner plexiform layer in the superficial capillary plexus (SCP). A) Non-flow angiogram of SCP. B) Capillary nonperfusion areas (mm^2^) were automatically calculated via OCTA in the SCP. C) Foveal avascular zone (FAZ) areas (mm^2^) were automatically calculated via OCTA in the SCP.

**Figure 4:**
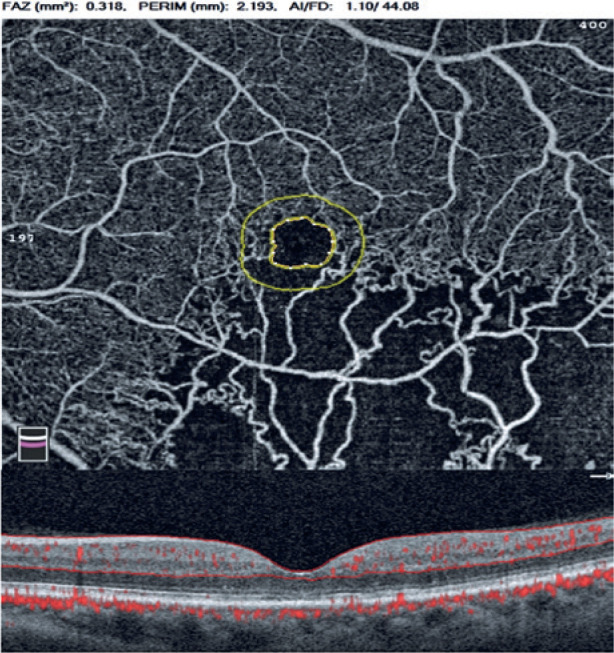
Foveal avascular zone (FAZ) assessment tool of optical coherence tomography angiography (OCTA) (above) and B-scan of the fovea (below). Segmentation was between the internal limiting membrane and the outer plexiform layer in full retinal vasculature. FAZ areas (mm^2^) were automatically calculated via OCTA in full retinal vasculature. PERIM (mm), perimeter of FAZ; AI, acircularity of FAZ; FD (%), foveal vessel density in a 300-_µ_m-wide region around FAZ (FD-300).

The FAZ area (mm^2^) in the SCP and CNP areas (mm^2^) was obtained via the non-flow assessment tool (Figure 3), and the FAZ area (mm^2^) in full retinal vasculature, FAZ perimeter (mm), acircularity index of FAZ, and foveal density (FD) (%) (foveal vessel density in a 300-µm-wide region around FAZ) were obtained via the FAZ assessment tool (Figure 4)^(8)^. OCTA assessments were performed after resolution of RVO-ME.

One experienced independent grader (P.K.) reviewed the OCTA images. Patients with poor image quality were excluded on the basis of the presence of at least one of the following criteria: low signal strength index <8; presence of ≥1 blink artifacts; poor fixation leading to motion or doubling artifacts; media opacity obscuring the view of the vasculature; and presence of cystoid macular changes resulting in disrupted retinal anatomic features and segmentation errors.

### Statistical analysis

The statistical analysis was performed using the SPSS software (Statistical Package for the Social Sciences) (version 18 for Windows; IBM Corp., Armonk, NY, USA). The normality of the data was confirmed using the Kolmogorov-Smirnov test (p>0.05). Pearson’s correlation coefficient was calculated to assess the correlation between the DRIL extent and OCTA parameters, including the superficial and whole-retina FAZ areas, CNP areas (non-flow), and vessel density of both SCP and DCP in the foveal, parafoveal, and perifoveal zones. A p-value <0.05 denoted statistically significant differences.

## RESULTS

Thirty-four eyes of 34 patients were included in this retrospective study. Clinical characteristics of the study patients are presented in table 1. The mean age of the pa tients was 58.27 ± 11.31 years, and 52.9% were males. Baseline mean BCVA was 0.77 ± 0.43 logMAR (20/117) and improved to 0.23 ± 0.32 logMAR (20/33) (p<0.001) at 6 months after initial treatment. Twenty-nine eyes were phakic, and five eyes were pseudophakic. There were two types of BRVO: superior temporal BRVO (70.0%) and inferior temporal BRVO (30.0 %). None of the patients received laser treatment.

**Table 1:** Demographic and clinical characteristics of the patients included in this study

Variable	
Age (years) Mean ± SD Range	58.27 ±11.31 (51-69)
Sex (n) Male (%) Female (%)	18 (52.9) 16 (47.1)
CCT, µm Mean ± SD Range	528 ± 24.66 (495-569)
Axial length, mm Mean ± SD Range	22.89 ± 0.12 (22.65-23.10)
Spherical equivalent, D Mean ± SD Range	0.18 ± 0.79 (-2.0-1.0)
1OP, mmHg Mean ± SD Range	14.94 ± 6.55 (8-19)

CCT= central corneal thickness; µm= micrometer; D= diopter; IOP= intraocular pressure; SD= standard deviation.

## Association of DRIL extent with OCTA parameters

The average measurement obtained by the two observers for the mean DRIL extent was 512.72 ± 238.47 (177-824) microns, and the mean CNP area was 4.98 ± 2.85 (1.34-8.71) mm^2^. There was a positive correlation between DRIL extent and CNP area (p<0.001, r=0.901). The mean FAZ, density, and non-flow assessment tool parameters of OCTA and their associations with DRIL extent and CNP areas are shown in table 2. Briefly, greater DRIL extent and CNP area were correlated with wider FAZ area (p=0.014 and p=0.036, respectively) in SCP and decreased FD (vessel density in 300 microns around the FAZ) (p=0.031 and p=0.022, respectively). They were also correlated with decreased vessel density in both SCP and DCP in the parafoveal and perifoveal regions (p<0.05 for all).

**Table 2:** Association of DRIL extent and CNP area with OCTA parameters

Variables	Mean ± standard deviation (range)	DRIL extent (µ)		CNP area (mm^2^)	
		P^*^	r^*^	P^*^	r^*^
Non-flow assesment tool					
FAZ area in SCP (mm^2^)	0.72 ± 0.20 (0.41-1.22)	**0.014**	**0.540**	**0.036**	**0.484**
FAZ assesment tool					
FAZ area in FRV (mm^2^)	0.30 ± 0.14 (0.05-0.57)	0.668	0.102	0.424	0.195
Perimeter (mm)	2.17 ± 0.51 (1.04-3.06)	0.150	0.343	0.881	0.037
Al	1.10 ± 0.03 (1.04-1.16)	0.237	0.285	0.846	0.048
FD (%)	46.42 ± 7.14(24.15-58.78)	**0.031**	**-0.496**	**0.022**	**-0.523**
Density assesment tool					
*Vessel density SCP %*					
Fovea	15.96 ± 4.54 (5.0-27.2)	0.238	-0.285	0.990	0.003
Parafovea	44.67 ± 5.80 (34.90-55.10)	**<0.001**	**-0.802**	**<0.001**	**-0.826**
*Perifovea*	46.22 ± 5.52 (38.10-54.90)	**<0.001**	**-0.858**	**<0.001**	**-0.939**
*Vessel density DCP %*					
Fovea	31.17 ± 9.72 (16.30-47.80)	0.309	-0.247	0.319	-0.241
Parafovea	47.32 ± 5.91 (37.60-58.30)	**<0.001**	**-0.721**	**<0.001**	**-0.809**
Perifovea	46.07 ± 11.56 (35.60-59.80)	**0.006**	**-0.618**	**0.003**	**-0.653**

* The mean FAZ, density, and non-flow assessment tool parameters of optical coherence tomography angiography and their Pearson’s correlation coefficients for the DRIL extent and CNP areas. Values in bold font are significant (p<0.05).

DRIL= disorganization of retinal inner layers; FAZ= foveal avascular zone; FRV= full retinal vasculature; AI= acircularity index; FD= foveal density; CNP= capillary nonperfusion; SCP= superficial capillary plexus; DCP= deep capillary plexus.

## DISCUSSION

In this study of patients with center-involved RVO-ME, DRIL was associated with capillary ischemia. Furthermore, wider DRIL extent was associated with wider FAZ area and decreased vessel density in both SCP and DCP.

OCTA is capable of independently revealing details in the macular capillary plexuses and the choriocapillaris^(9)^. The device divides the intraretinal structures of major capillary networks into segments: SCP, between the internal limiting membrane (ILM) and the inner plexiform layer, and DCP, between the inner nuclear layer and the outer plexiform layer. Additionally, the device automatically detects the borders of the FAZ and calculates its area, acircularity index of FAZ, and perimeter. In center-involved ME, secondary to any cause of retinal diseases, segmentation errors and disrupted retinal anatomic features may occur, altering the results of these calculations. In this study, we assessed the association between DRIL extent and OCTA parameters after resolution of RVO-ME.

FD is a vessel density parameter within 300 microns around the FAZ in the FAZ assessment tool of OCTA. The device automatically detects the borders of the FAZ, draws another circle around the FAZ at a distance of 300 microns, and calculates the vessel density in this region^(8)^. In our study, FD was significantly associated with the DRIL extent and CNP area (p=0.031 and p=0.022, respectively). The density assessment tool of OCTA also calculates the FD in both SCP and DCP. The device calculates the vessel density within a 1-mm diameter of the fovea-centered circle. However, these two parameters differ in that there was no significant correlation between the vessel density in the fovea in either of the macular capillary plexuses and DRIL extent and CNP area (p>0.05 for all). We hypothesized that this difference occurs because of the widened FAZ area in BRVO. As long as the FAZ widens, the FD will not be affected, because the device always measures the vessel density between the two circles (Figure 4). Nevertheless, in the vessel density assessment tool of OCTA, the circles are constant. The device automatically inserted three fovea-centered circles on the macula with diameters of 1, 3, and 6 mm (Figure 2). As the FAZ widens, the vessel density of the fovea in both SCP and DCP decreases. As a result, we suggest that, during the evaluation of the FD, the FD parameter in the FAZ assessment tool of OCTA should be used instead of the density assessment tool.

In our study, we found that the DRIL extent and CNP areas were associated with the FAZ area when using the non-flow assessment tool (p=0.031 and p=0.022, respectively). However, this association was not observed when using the FAZ assessment tool (p=0.668 and p=0.424, respectively). We hypothesized that this difference is caused by the position of segmentation lines. In the non-flow assessment tool of OCTA, both the CNP areas and FAZ area were calculated in SCP (the segmentation lines were between the ILM and inner plexiform layer). However, in the FAZ assessment tool, the FAZ area was calculated in full retinal vasculature (the segmentation lines were between the ILM and outer plexiform layer); however, the CNP area was calculated in SCP (Figures 2 and 3). Coscas et al.^(10)^ reported that the DCP was more severely affected than the SCP in RVO.

The choroid principally provides nourishment of the cells within the FAZ. However, following RVO, the choroid is unable to sufficiently oxygenate the inner retina, leading to imbalance between the oxygen supply and consumption^(11)^. This process affects the size of FAZ and visual function^(11)^. Parodi et al.^(12)^ compared the FAZ areas of patients with BRVO and controls via FFA. The mean FAZ area was greater in eyes with BRVO (0.56 ± 0.34 mm^2^) compared with that in controls. The investigators reported a significant correlation between BCVA and FAZ enlargement. Samara et al.^(13)^ evaluated 17 eyes with BRVO and compared FAZ area measurements between the eye with BRVO and the fellow normal eye. They reported that the FAZ area in the BRVO eye was significantly enlarged in the DCP^(13)^. Kashani et al.^(14)^ showed that OCTA is capable of evaluating and managing the macular complications of RVO. Balaratnasingam et al.^(11)^ showed a significant correlation between the FAZ and DRIL; however, they did not stratify capillary networks into SCP and/or DCP because of segmentation errors. In our study, the FAZ area was 0.72 ± 0.20 (0.41-1.22) mm^2^ in the SCP and was also significantly associated with DRIL (p=0.014).

**Figure 5:**
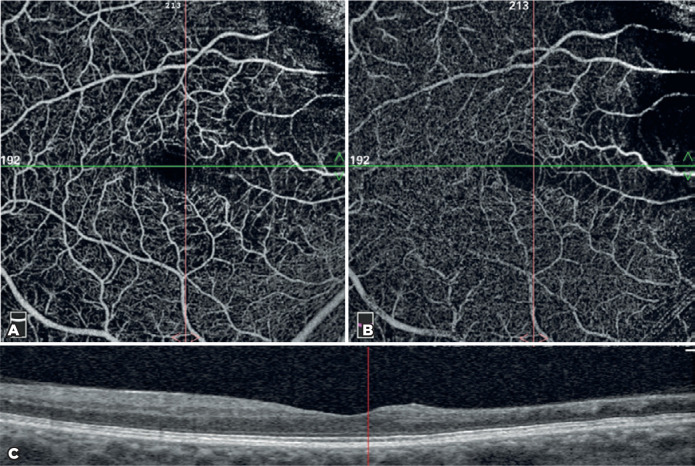
Evaluation of the same patient shown in figure 1 through optical coherence tomography angiography (OCTA). Capillary nonperfusion areas comply with areas of disorganization of the retinal inner layers (DRIL). A) Superficial capillary plexus segmentation. B) Deep capillary plexus segmentation. C) B-scan of the fovea.

DRIL represents the destruction of cells within inner retinal layers, and it is thought to be associated with regions where synaptic connections of amacrine, bipolar, and horizontal cells within the inner retina have been disrupted^(4)^. Nourishment of the inner retina is predominantly provided by retinal circulation^(15^,^16)^. Therefore, disruption of the perfusion of the inner retina caused by RVO will result in structural changes that manifest as DRIL on SD-OCT. Grewal et al. reported that inflammation and ischemia are the main potential drivers in the pathogenesis of DRIL in uveitic CME^(6)^. They also hypothesized that DRIL may be a generic finding of tissue damage due to ischemia; similar loss of retinal lamination has also been observed following RVO^(6)^. Moreover, Moein et al. investigated the foveal vascular architecture in patients with and without DRIL after resolved diabetic ME and reported that the CNP areas complied with those of DRIL^(17)^. In our study, we found a positive correlation between the DRIL extent and CNP area (p<0.001, r=0.901) and showed that areas of capillary ischemia correspond to areas of DRIL in eyes with center-involved ME associated with RVO (Figure 5). Our findings also indicated that DRIL may be correlated with capillary ischemia. Additionally, the DRIL extent was correlated with decreased vessel density parameters in the parafoveal and perifoveal regions in both SCP and DCP (p<0.05 for all).

We acknowledge several limitations of the study. First, this was a retrospective study involving a limited number of patients. Prospective studies with larger sample sizes are warranted to confirm the findings of this investigation. Second, the DRIL extent was measured manually; however, we attempted to minimize the likelihood of bias by using two observers. In the future, the capabilities of automated software may be enhanced for the objective measurements of the DRIL extent. Finally, histologic and pathologic evaluations of DRIL would deepen our knowledge regarding its pathophysiology. Strengths of the study include the quantitative assessment of the CNP area and its association with DRIL via OCTA.

In conclusion, DRIL appears to be correlated with capillary ischemia in RVO. The DRIL extent was strongly correlated with the CNP area. This finding may support the notion that the DRIL extent can be used as an easily obtainable and important surrogate marker of capillary ischemia.
